# Different Strategies for the Identification of SARS-CoV-2 Variants in the Laboratory Practice

**DOI:** 10.3390/genes12091428

**Published:** 2021-09-16

**Authors:** Federico Anaclerio, Rossella Ferrante, Domitilla Mandatori, Ivana Antonucci, Matteo Capanna, Verena Damiani, Pamela Di Tomo, Roberto Ferrante, Marianna Ranaudo, Vincenzo De Laurenzi, Liborio Stuppia, Simone De Fabritiis

**Affiliations:** 1 Center for Advanced Studies and Technology (CAST), G.d’Annunzio University of Chieti-Pescara, 66100 Chieti, Italy; federico.anaclerio@hotmail.it (F.A.); rossellaferrante@yahoo.it (R.F.); i.antonucci@unich.it (I.A.); matteo_capanna@live.it (M.C.); verena.damiani@unich.it (V.D.); pamela.ditomo@unich.it (P.D.T.); ferranterr@outlook.it (R.F.); mar.ranaudo@gmail.com (M.R.); delaurenzi@unich.it (V.D.L.); stuppia@unich.it (L.S.); simone.defabritiis@unich.it (S.D.F.); 2 Department of Medical Oral and Biotechnological Sciences, School of Medicine and Health Sciences, G.d’Annunzio University of Chieti-Pescara, 66100 Chieti, Italy; 3 Department of Psicological Health and Territory Science, School of Medicine and Health Sciences, G.d’Annunzio University of Chieti-Pescara, 66100 Chieti, Italy; 4 Department of Innovative Technologies in Medicine and Dentistry, School of Medicine and Health Sciences, G.d’Annunzio University of Chieti-Pescara, 66100 Chieti, Italy; 5 Department of Medicine and Aging Sciences, School of Medicine and Health Sciences, G.d’Annunzio University of Chieti-Pescara, 66100 Chieti, Italy

**Keywords:** SARS-CoV-2 variants, qRT-PCR, NGS, Sanger, tracking, laboratory practice

## Abstract

A considerable effort has been devoted in all countries to react to the COVID-19 pandemic by tracing infected individuals, containing the spread of the disease, identifying therapies, and producing and distributing vaccines. Currently, a significant concern is the appearance of variants of the virus that may frustrate these efforts by showing increased transmissibility, increased disease severity, reduced response to therapy or vaccines, and ability to escape diagnosis. All countries have therefore devoted a massive attempt to the identification and tracking of these variants, which requires a vast technological effort to sequence a large number of viral genomes. In this paper, we report our experience as one of the Italian laboratories involved in SARS-CoV-2 variant tracing. We summarize the different approaches used, and outline a potential model combining several techniques to increase tracing ability while at the same time minimizing costs.

## 1. Introduction

Over one year after the appearance of the first cases in China, the COVID-19 pandemic still represents a dramatic health emergency worldwide. Despite the enormous efforts devoted to the production of vaccines, which have already been provided to a huge number of individuals, significant concern has been raised due to the identification of several variants in the SARS-CoV-2 genome which result in changes in the infectivity, response to vaccines and lethality of the virus [1,2]. Indeed, the rapid spreading of some of these variants has shown that reinfections are possible in previously infected patients as well as in vaccinated subjects [3,4].

Thus, identifying the SARS-CoV-2 variants and tracking their diffusion throughout the world plays a crucial role in controlling the pandemic. Based on the effect on infectivity, lethality and response to therapy, Tte US government SARS-CoV-2 Interagency Group (SIG) developed a Variant Classification scheme that defines three classes of SARS-CoV-2 variant: Variants of Interest (VOI), Variants of Concern (VOC) and Variants of High Consequence (VHC).

VOIs are characterized by genetic markers associated with changes to receptor binding, reduced neutralization by antibodies generated against previous infection or vaccination, reduced efficacy of treatments, potential diagnostic impact, or predicted increase in transmissibility or disease severity. Examples of VOIs are represented by B.1.525, B.1.526, B.1.526.1, B.1.617, B.1.617.1, B.1.617.3 and P.2.

On the other hand, VOCs are characterized by an increase in transmissibility, more severe disease (increased hospitalizations or deaths), a significant reduction in neutralization by antibodies generated during previous infection or vaccination, reduced effectiveness of treatments or vaccines, or diagnostic detection failures [5,6,7]. B.1.1.7, P.1, B.1.427, B.1.429, B.1.351 and B.1.617.2 are typical examples of VOCs.

Variants of High Consequence (VHC), those for which preventative measures or medical countermeasures could have significantly reduced effectiveness relative to previously circulating variants, have also been hypothesized. However, so far, no SARS-CoV-2 variant that rises to the level of high consequence has been reported.

Being characterized by different nucleotide changes in the viral genomic sequence, SARS-CoV-2 variants can be identified by different techniques; in the majority of cases different variants are not detected by routine Real-Time PCR approaches used for the molecular diagnosis of viral infection. However, some specific PCR assays, by including the *S* gene among the analyzed viral targets, can identify variants carrying the Δ69–70 deletion, found in some lineages including the B.1.1.7, currently the most common variant throughout Europe [8]. Indeed, when using some of the presently available commercial kits, the presence of this 6 bp deletion results in the absence of *S* gene amplification while the amplification of other targets is not affected. Therefore, these assays can be used as a screening test to track this VOC. In addition, other PCR assays have been recently developed explicitly to identify the presence of other common mutations, in addition to the Δ69–70. They can be very useful in screening for other VOCs, such as the P.1 or the B.1.351.

Sequencing part or all of the genome remains the only way to confirm the presence of known mutations or to identify new ones. In general, sequencing of the *S* gene alone by the Sanger method can be sufficient to identify most of the known variants, all these being characterized by the presence of specific mutations in this gene. However, knowledge of the presence of mutations in other viral genes can be helpful and should not be neglected. Whole sequencing of the viral genome by Next-Generations Sequencing (NGS) therefore represents the gold standard for the study of variants, allowing identification and detection of all possible variants of the SARS-CoV-2 sequence. However, this approach can be quite expensive and time-consuming, and the technology is not available in all laboratories.

Here we report our experience in analysing SARS-CoV-2 variants using the different strategies discussed above to suggest the best practices to be used to detect the presence and prevalence of SARS-CoV-2 variants.

## 2. Materials and Methods

### 2.1. Patients

From April 2020 to April 2021 about 150,000 nasopharyngeal swabs were analyzed in the lab of Molecular Genetics–COVID-19 Diagnosis of the Center of Advanced Studies and Technology (CAST), G. d’Annunzio University of Chieti-Pescara, Italy. These samples mainly consisted of hospitalized patients being tested for the purpose of COVID-19 diagnosis. However, molecular diagnosis of SARS-CoV-2 infection was also offered as a screening service to healthcare workers and employees of different local companies.

Selection of SARS-CoV-2 cases to be investigated for the presence of specific variants was carried out according to the recommendation of the Italian Istituto Superiore di Sanità (ISS) based on the presence of one of the following features:

1. Absence of amplification of the *S* gene.
2. Trips abroad from geographic areas known to be characterized by VOCs.
3. Suspected SARS-CoV-2 reinfection.
4. Clusters with a high prevalence of infection.
5. Infection in previously vaccinated subjects.

In addition, starting from February 2021, other samples were randomly sequenced to be included in the national monthly surveys of the ISS based on sequencing of a representative percentage of all novel positive samples on a specific day.

### 2.2. RNA Extraction and qRT-PCR

For routine analysis, nasopharyngeal specimens in universal transport medium (UTM) were opened under biosafety hood, and 200 μL were used for RNA extraction using MagMAX Viral/Pathogen II (MVP II) Nucleic Acid Isolation Kit and the automated KingFisher magnetic particle processor (Thermo Fisher Scientific, Waltham, MA, USA), as indicated in the manufacturer’s instructions. Then, 10 μL of the extracted RNA underwent real-time reverse transcription polymerase chain reaction (qRT-PCR) using the TaqPath™ COVID-19 CE-IVD RT-PCR Kit assay (Thermo Fisher Scientific, Waltham, MA, USA) following the kit’s instruction. The QuantStudio 5 Real-Time PCR System assay (DX) was used for qRT-PCR analysis (Thermo Fisher Scientific, Waltham, MA, USA). The TaqPath™ assay targets three different viral genomic regions: *ORF1ab, N* and *S* genes. A valid negative result for SARS-CoV-2 was determined by amplification of the only MS2 internal control. A specimen was considered positive in the presence of amplification of at least two of the three target genes. Particularly, positive samples were classified as “S positive” when all the three targets were detected or when only two targets were detected, including S. On the other hand, samples were defined as “S negative” when the S target was not amplified.

### 2.3. Variants qRT-PCR

Two hundred and thirty-two positive specimens were further analysed with two qualitative qRT-PCR commercial kits: Allplex^TM^ SARS-CoV-2 Variants I Assay (Seegene, Korea) and COVID-19 Variant Catcher CE-IVD kit (Clonit, Italy), that allows the simultaneous identification of the *S* gene Δ69–70, E484K and N501Y mutations for discrimination of SARS-CoV-2 (NC_045512.2) from other SARS-CoV-2 strains B.1.1.7, B.1.351 and P.1. The CFX96 Touch™ Real-Time PCR Detection System (BIO-RAD, USA) was used as the instrument for these analyses.

### 2.4. Sanger Sequencing

RNA samples of six subjects were reverse transcribed using SuperScript IV VILO Master Mix (Thermo Fisher Scientific, Waltham, MA, USA). Twelve pairs of M13-tagged primers were used in the amplification step by PCR to cover the whole S viral gene, using the BigDye Direct PCR Master Mix (Thermo Fisher Scientific, Waltham, MA, USA). Once the PCR step is complete, the cycle sequencing reaction was carried out by directly adding the BigDye Direct Sequencing Master Mix and BigDye Direct M13 Forward or Reverse primer (Thermo Fisher Scientific, USA) in the PCR product. The sequencing cleanup was performed using the NucleoSEQ kit for dye terminator removal (Macherey-Nagel, Dueren, Germany) and finally subjected to capillary electrophoresis using the SeqStudio^TM^ Genetic Analyzer (Applied Biosystems, Waltham, MA, USA). The raw reads were analyzed using Sequence Scanner v2.0 to generate a QC report. The adequate sequencing traces were exported as FASTA files. The sequences generated were verified using BLAST alignment software with the reference genome (NC_045512.2).

### 2.5. Next-Generation Sequencing (NGS)

**Figure 1:**
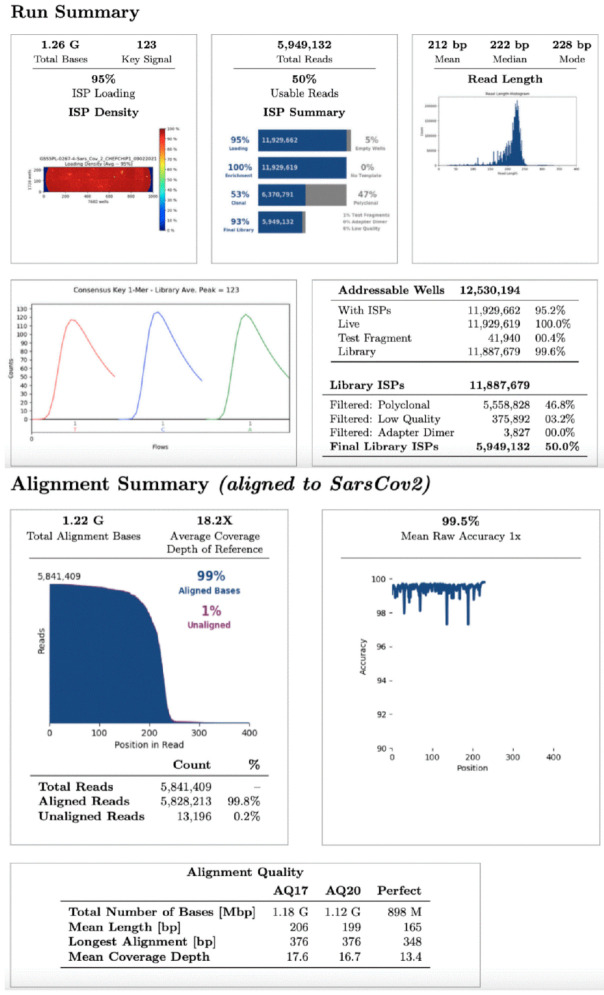
**NGS run summary.** Representation of an NGS data analysis report about SARS-CoV-2 sequencing.

For whole viral genome sequencing, total RNA was reverse transcribed using Invitrogen SuperScript VILO^TM^ cDNA Synthesis Kit (Thermo Fisher Scientific, Waltham, MA, USA). One hundred and seven samples were analyzed in eight different sequencing runs using the Ion Torrent S5 system (Thermo Fisher Scientific, Waltham, MA, USA) after library preparation, consisting of fragmentation and adapter ligation onto the PCR products and clonal amplification. cDNA libraries were then prepared using the Ion AmpliSeq SARS-CoV-2 Research Panel (Thermo Fisher Scientific, Waltham, MA, USA). After quantification of cDNA libraries with Real-Time Step One PCR System (Thermo Fisher Scientific, Waltham, MA, USA), the prepared samples of ion sphere particles (ISP) were loaded onto an Ion 520™ chip with the Ion Chef (Thermo Fisher Scientific, Waltham, MA, USA). Sequencing was performed using the Ion S5™ sequencing reagents (Thermo Fisher Scientific, Waltham, MA, USA). The Torrent Suite 5.14.0 platform and specific plugins were used for NGS data analysis. All analysed sequences showed an alignment accuracy of over 96% and a base coverage over 20× (Figure 1). The pangolin software was used for the assignment of SARS-CoV-2 lineages. All sequences were then submitted as FASTA files on gisaid.org, which provides open access to genomic data on SARS-CoV-2.

## 3. Results

The large majority of positive cases detected from April 2020 to November 2020 showed the amplification of *S, N* and *ORF1ab* genes. However, only a limited number of cases were characterized by the presence of two of these targets (*S* + *N*, *S* + *ORF1ab* or *N* + *ORF1ab*) and in no cases were infection clusters showing this feature found.

**Figure 2:**
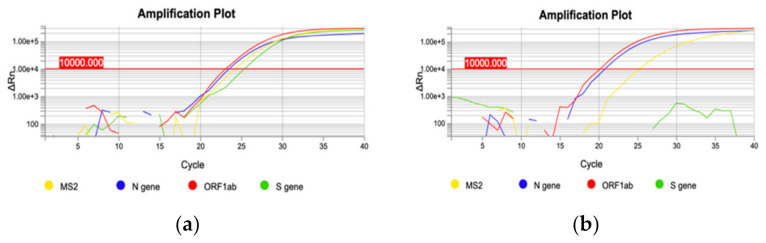
Two representative qRT-PCR amplification plots. (**a**) amplification of three targets: *N* gene, *ORF1ab* gene and *S* gene (blue, red and green curve, respectively); (**b**) *S* gene deletion: only *N* gene and *ORF1ab* gene targets are amplified (blue and red curve, respectively).

**Figure 3:**
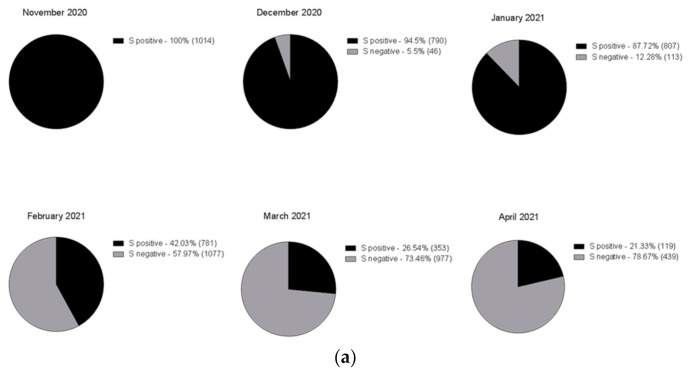

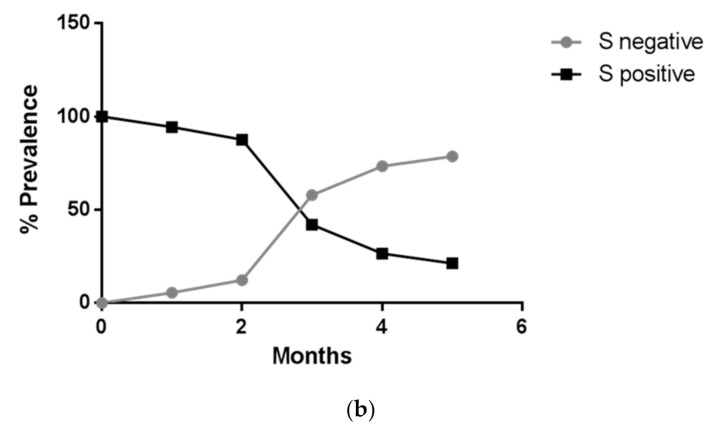
Time course of the number of positive cases with three genes amplified (black) and with two genes (grey), shown as (**a**) pie chart (absolute frequency percentage) or (**b**) line graph (prevalence percentage).

On December 22nd, a cluster of 31 individuals (represented by subjects detained in a local prison) all showing amplification of only the *N* and *ORF1ab* targets immediately captured our attention (Figure 2). In fact, a few days prior it had been reported that the lack of amplification of the *S* gene target with some of the available commercial kits (including the one we were using at the time) was a typical feature of the Δ69–70 variant, present in the B.1.1.7 lineage. We therefore adopted different strategies to better characterize all the samples showing the Δ69–70, as described below. Sequencing of samples from this cluster showed, as expected, that all viruses belonged to the same strain; however, it was not the suspected B.1.1.7 lineage, but rather a different one, B.1.258, which also presents the Δ69–70 deletion. Only a few other cases of this particular strain were detected in the following months outside the cluster. However, sequencing of a few other cases with a lack of S amplification from subjects outside the jail cluster revealed the presence of the B.1.1.7 variant in one case. As it turned out, this case belonged to the first real cluster of this variant in the region, which started in a small village. From this time the B.1.1.7 variant began spreading in the region, as it did throughout the rest of Italy. Figure 3 shows the rapid spreading of the B.1.1.7 lineage that reached 78% of positive cases in April 2021. Tracking the variant based on the lack of S amplification followed by confirmation via sequence analysis, in cases from different areas and clusters, proved to be a very useful, rapid and relatively cheap approach to variant mapping.

Following the experience described above, we sought other methods that allowed a fast screening of positives to reduce the number of samples that underwent partial or complete sequencing. As a result, we used variant-specific qRT-PCR tests with probes designed to detect Δ69–70, N501Y and E484K mutations on 232 positive cases detected between February 2021 and April 2021, based on ISS recommendations for variant surveillance. The combination of these mutations led us to suspect the presence of several VOCs, namely: B.1.1.7, B.1.351 or P.1.

**Figure 4:**
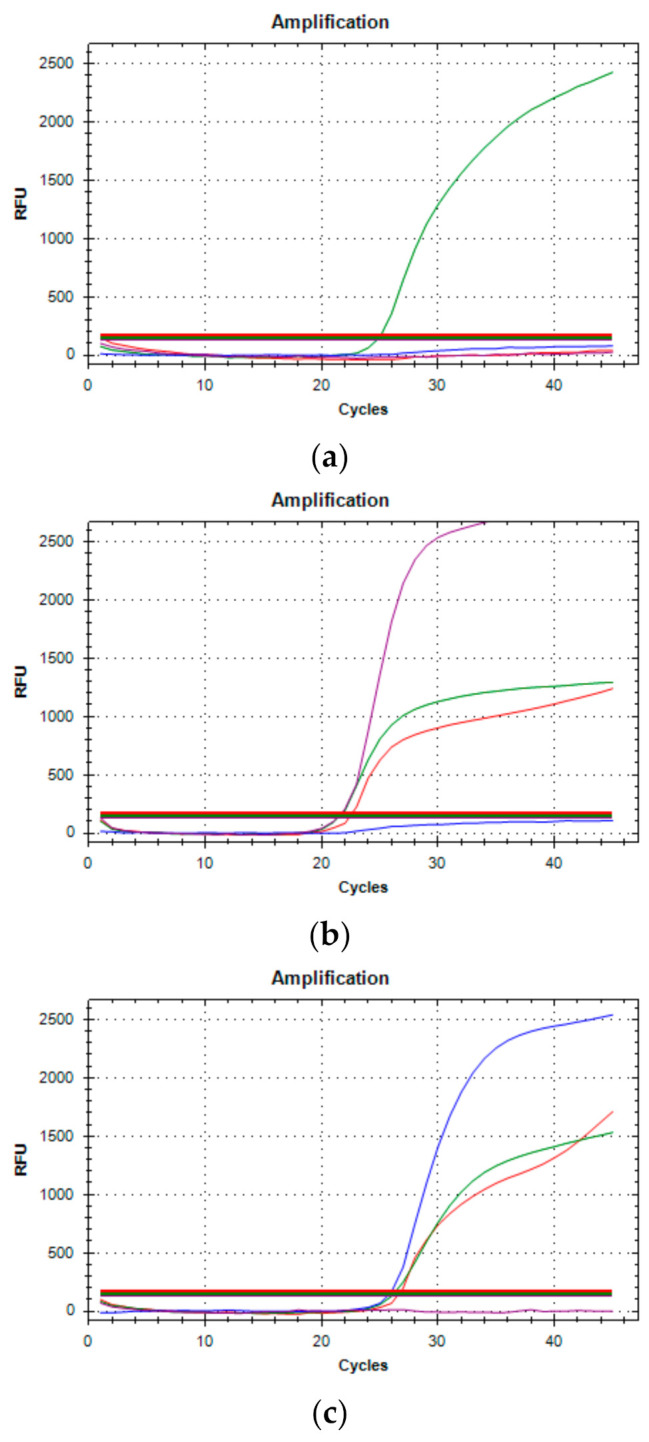
Three typical amplification results of Variants qRT-PCR. (**a**) The presence of only one green amplification curve indicates that wild type (WT) sequences of SARS-CoV-2 have been amplified. (**b**) Purple, red, and green curves occurring simultaneously (69-70del, N501Y mutated and E484K WT, respectively) determine for the B.1.1.7 variant. (**c**) Blue, red and green curves (E484K mutated, N501Y mutated and 69-70del WT, respectively) detect the B.1.351 or P.1 variant.

We were able to assign 100 cases (43.10%) as B.1.1.7, as all showed both the Δ69-70del and N501Y mutations; sequencing of the *S* gene by Sanger or the whole viral genome by NGS of a representative number of samples confirmed the strain identity. Two of the positive cases showed the presence of the E484K mutation alone and could not, therefore, be assigned to one of the different VOCs that carry this mutation. Sanger sequencing of the *S* gene could not assign these cases to a specific strain; therefore, the sequencing of the entire genome by NGS allowed the identification of the rare C.11 strain. One additional sample showed the presence of the E484K and N501Y mutations suggestive of at least two variants (B.1.351 and P.1). Sequencing by NGS confirmed that the case belonged to the P.1 strain. The remaining 93 samples (40.10%) showed no mutations detected by the variants’ specific qRT-PCR kits (Figure 4). Some of these were then subjected to sequence analysis, resulting in our identifying several different strains, all previously described elsewhere and none of which was classified as a VOI.

Finally, 36 (15.51%) of the samples were not amplified, showing a lower sensitivity of this PCR approach than the PCRs used for diagnosis.

In parallel to this approach, we participated in a number of surveys promoted by the ISS, as did all Italian regions during this period. These exercises consisted of sequencing a representative percentage of all positive samples from a specific day. A complete list of all strains identified by the lab using the different approaches is reported in Table 1. It confirms that most cases belong to the B.1.1.7 strain and the second most frequent was B.1.177 (19.7% of positive samples).

## 4. Discussion

The progressive diffusion of different SARS-CoV-2 variants worldwide highlighted the need to develop specific tools aimed at identifying and tracing their distribution and spreading throughout the population. While sequencing of the part (namely the *S* gene) or the entire genome in many samples representative of the entire population is probably the only way to identify new variants of potential clinical interest, other approaches can be used to trace known variants [9]. Simply paying attention to abnormal amplification patterns (i.e., lack of *S* amplification) might raise awareness with respect to a particular group of samples. Once verified, this abnormal pathway is characteristic of a particular variant and used to trace it throughout the population.

Variant specific qPCR has been developed by many companies and is updated continuously. Although in general not adequate as a diagnostic test, this appears to be extremely useful as a cheap screening tool that allows tracing of specific variants, thus reducing the number of sequence analyses [10].

Indeed, using this approach may significantly reduce the number of samples that require sequencing and dedicate most of the sequencing effort to those samples that do not carry known mutations of interest, thus leading to the identification of novel strains. Moreover, this approach allows rapid tracing of known variants and the possibility of alerting local authorities to their spreading almost in real-time, thus providing better control of the pandemic.

Sequencing of the *S* gene can be a valuable tool for confirmation of known variants following screening by PCR as described above. However, the gold standard for the identification of new variants remains sequencing of the entire viral genome by NGS, since mutations outside the *S* gene, able to change the infectivity of the virus and the prognosis of the infection, have also been reported [11,12,13]. Indeed, mutations associated with prognosis changes have been described in the *ORF8*, *ORF3a*, *ORF6*, and *N* genes; for example, deletion of 382 nucleotides (D382) within *ORF8* has been associated with a milder infection and a lower concentration of proinflammatory cytokines [14]. Frameshift/nonsense mutations of *ORF6* are responsible for the upregulation of interferon type I (IFN) and more severe disease [15,16].

It should be mentioned that in the case of a large number of samples, the costs of NGS sequencing are dramatically reduced and can be competitive with Sanger sequencing. In these cases, the use of this latter method should be limited to confirmation of mutations within the *S* gene when a limited number of cases are analysed [17].

When sequencing technology and particularly NGS is not available, as in many small diagnostic centres, we believe that these labs should set up alternative screening methods such as variant specific PCRs to perform rapid screenings of the different VOCs, and that they should be connected with other labs able to perform NGS analysis on an extensive series of samples. In the context of the need for identification and tracking of SARS-CoV-2 variants in a given territory this kind of organization, involving several spokes performing infection diagnosis and able to report suspected VOCs to a few hubs able to performing Sanger and mainly NGS analysis, is necessary. An example was represented by the recent diffusion of the B.1.617.2 variant. In the period between June and July 2021, among all positive samples (347 total cases) analyzed in our laboratory, 48.7% resulted S negative, while the others showed three target amplification (51.3%). Based on our approach, 43 specimens of this 51.3% were randomly sequenced by NGS or Sanger, immediately revealing the B.1.617.2 variant in 100% of the cases, suggesting a more rapid spread compared to B.1.1.7 variant.

## 5. Conclusions

In conclusion, this confirms that such organization is more effective than random analysis of positive samples by “spoke” labs, allowing rapid identification and tracking of VOCs.

## Figures and Tables

**Table 1:** Lineages identified and relative number of cases and percentage from December 2020 to April 2021.

Lineages	Number of Cases	Percentages
B.1.1.7	51	47.7%
B.1.177	21	19.7%
B.1.1.420	10	9.4%
B.1.258	8	7.5%
B.1.177.75	4	3.7%
B.1.160	3	2.8%
C.11	2	1.9%
B.1.1	2	1.9%
P.1	1	0.9%
B.1	1	0.9%
B.1.177.6	1	0.9%
B.1.177.8	1	0.9%
B.1.177.33	1	0.9%
B.1.177.83	1	0.9%

## Data Availability

Data available on request.
